# Correlation of 360-degree Surface Mapping *In Vivo* Bioluminescence with Multi-Spectral Optoacoustic Tomography in Human Xenograft Tumor Models

**DOI:** 10.1038/s41598-018-21668-4

**Published:** 2018-02-20

**Authors:** Andrew Brannen, Matthew Eggert, Matthias Nahrendorf, Robert Arnold, Peter Panizzi

**Affiliations:** 1 Department of Drug Discovery and Development, Harrison School of Pharmacy, Auburn University, 247 Pharmacy Research Building, 720 South Donahue Dr., Auburn, AL 36849 USA; 2 Auburn Laboratory for Imaging Animal Systems, College of Veterinary Medicine, Auburn University, 403 Green Annex, Auburn, AL 36849 USA; 3 Center for Systems Biology, Massachusetts General Hospital & Harvard Medical School, 185 Cambridge Street, Boston, MA 02115 USA

## Abstract

Pre-clinical monitoring of tumor growth and identification of distal metastasis requires a balance between accuracy and expediency. Bioluminescence imaging (BLI) is often used to track tumor growth but is primarily limited to planar 2-dimensional (2D) imaging. Consistent subject placement within a standard top-mounted, single-detector small animal imager is vital to reducing variability in repeated same-animal measures over time. Here, we describe a method for tracking tumor development using a multi-angle BLI and photo-acoustic workflow. We correlate serial caliper measurements and 2D BLI to 360° BLI and photo-acoustic datasets for the same animals. Full 360° BLI showed improved correlations with both volumes obtained from caliper measurements and photo-acoustic segmentation, as compared to planar BLI. We also determined segmented tumor volumes from photo-acoustic datasets more accurately reflects true excised tumors’ volumes compared to caliper measurements. Our results demonstrate the distinct advantages of both 360° surface mapping by BLI and photo-acoustic methodologies for non-invasive tracking of tumor growth in pre-clinical academic settings. Furthermore, our design is fully implementable in all top-mounted, single-detector imagers, thereby providing the opportunity to shift the paradigm away from planar BLI into rapid BLI tomography applications.

## Introduction

Routine tracking of tumor growth in mice is an essential component of most pre-clinical cancer studies. In the past, caliper measurements have been the primary tool researchers had for monitoring the development of subcutaneously implanted tumors over time. Yet these methods have high relative standard deviations caused by the inability to circumvent the entire tumor in a living animal, so that tumor size is approximated rather than truly defined^1,2^. To address this problem, subcutaneously implanted tumors are often presumed to be ellipsoidal in shape, with volumes often calculated with the assumption that the z-axis is equal to the shorter of the x/y axis dimensions^3–5^. As a result, inherently inconsistent tumor morphology causes noisy tumor development progress curves and, ultimately, higher animal usage to reach statistical significance in comparative studies. Despite these issues, calipers remain the primary standard of tumor measurement in many cancer studies.

The advent of contrast-enhanced micro-computed tomography (CT) and magnetic resonance imaging (MRI) has revolutionized tumor volume determination along the continuum of disease progression^5,6^. However, both modalities have inherent limitations. CT requires ionizing radiation and relies on exogenous agents to deliver the soft tissue contrast needed to define tumor borders and vascular supply networks. MR imaging requires longer acquisition times and increased budgetary considerations, especially for routine measurements of tumor size in large cohorts of animals^3,7^. Furthermore, most academic settings and vivaria have limited access to these technologies, thereby reducing both modalities’ utility for assessing tumor volume^7^. Using surrogate signals to approximate tumor volumes is an attractive alternative approach to quantifying tumor size and response to therapy. Surrogate signal techniques include optical imaging approaches, such as bioluminescence, fluorescence (fluorescence reflectance imaging (FRI) and fluorescence molecular tomography (FMT)), and radiologic methods such as single positron emission computed tomography (SPECT) and positron emission tomography (PET). Other technologies such as photo-acoustic and hybrid photo-acoustic/ultrasonic imaging use the absorptive properties of endogenous chromophores for contrast while facilitating exogenous probe detection to measure signal deep within animals^8,9^.

Bioluminescence imaging (BLI) is another popular alternative to track relative tumor growth and anti-cancer treatment efficacy^10^. Traditional *in vivo* BLI is a planar projection that collapses all light production within a given space into a singular 2-dimensional or flat image and has no capacity to equate volumes or assign depth to these measures. Yet BLI remains an attractive imaging modality because, especially in cancer studies, animals can be implanted with a diverse range of luciferase-expressing cell-lines and imaging luciferase-positive cancer cells would only require intraperitoneal (IP) administration of the small-molecule substrate luciferin. Since luciferase is not found in mammals, light production is limited to sites where luciferin is oxidized by intracellular luciferase contained with these bio-engineered cancer cells. This affords BLI a distinct advantage over calipers as tumors located within the body cavity can be monitored via their total light production, including metastatic tumors that localize to distance sites such as the lungs or brain. Despite its appeal, light production in BLI is governed by the availability of aforementioned exogenous luciferin substrate, adenosine triphosphate (ATP), and molecular oxygen, all of which must be delivered to luciferase-expressing cancer cells by highly developed vascular or vascular-mimicking networks ingrained in developing tumors^11–13^. BLI is also limited by the physical location of the bioluminescent signal source within the body relative to the detector.

In addition to influencing fuel delivery to the light-producing cellular engine, the physical location of the bioluminescent source also profoundly impacts the perceived cellular density implied by the overall intensity readings collected from an image collected at a single vantage point. The confounding factor is that as light moves through the animal, there is a scattering effect due to the inherent properties of tissue as a non-homogenous medium for light propagation and diminished intensity caused by the absorptive properties of blood, fat, and muscle^14^. These tissue-scattering considerations complicate longitudinal BLI studies, as animal positioning needs to be consistent among all time points in a given dataset for optimal results^5^. To address positional bias, researchers often manually orient the animal and acquire multiple images in an attempt to find the optimal orientation for each time point. However, this process is often laborious and subjective, particularly when attempting to localize metastatic sites at a distance from the primary tumor site. The magnitude of BLI signal is related to both the size (*i*.*e*. density) of luciferase expressing cells and depth of these masses within the animal. Reconstructing diffuse BLI projections back into the original source by use of tomographic algorithms (so called BLI tomography or BLT) has been met with some success, but there is no standard convention to move from BLI to BLT^15–25^. The advantage our 360° surface mapping for BLI presented here is that it would provide consistent datasets to evaluate the comparative merits of different BLT algorithms moving forward.

The goals of this study were (1) to develop a method for eliminating positional bias in order to improve correlations between longitudinal BLI and tumor volume, (2) to create a standardized and cost-effective method for labs to generate 360° surface mapping of BLI datasets, and (3) to validate multi-spectral optoacoustic tomography (MSOT) for quantifying tumor volumes. To this end, we tracked the growth of subcutaneous prostate cancer xenografts via the *Mouse Imaging Spinner* (MiSpinner), our developed-in-house prototype for acquiring 360° *in vivo* BLI, in athymic mice over a 9-week time course. We evaluated standard planar BLI, MiSpinner-determined optimal angle BLI, and area under the curve (AUC) of 360° BLI and correlated these measures with volumes determined by digital caliper measurements, volumetric MSOT segmentations, and *ex vivo* digital caliper measurements. Our results indicated that MiSpinner-based BLI data better correlated with tumor volumes as compared to planar static BLI. Further, we demonstrated that volumes determined from photo-acoustic imaging using the MSOT more accurately reflected the true tumor volumes as measured *ex vivo* by digital calipers.

## Results

### MiSpinner System Components and Assembly

**Figure 1:**
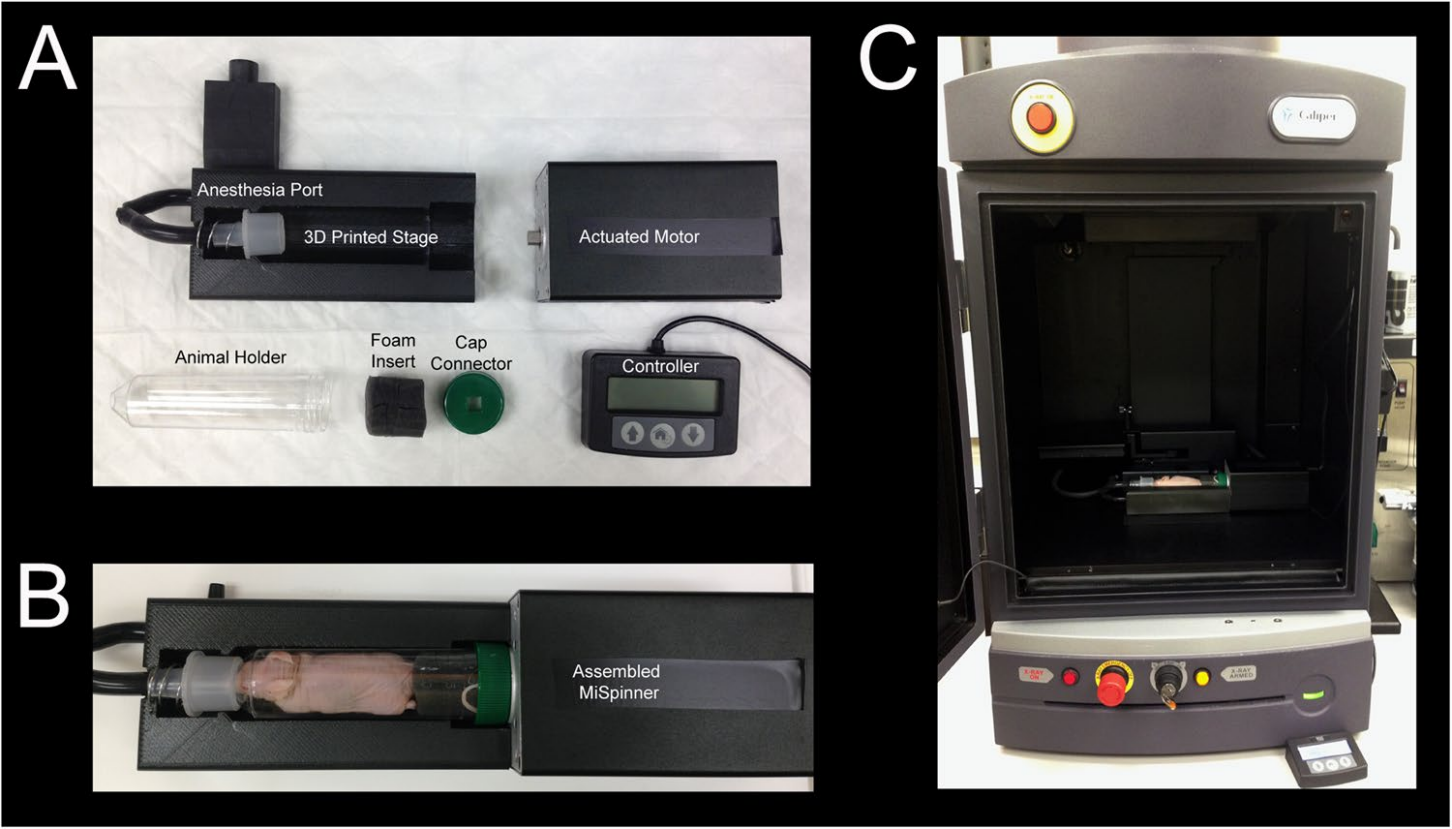
(**A**) The *MiSpinner* comprises a step motor attached to a custom 3D-printed stage with an integrated gas anesthesia port. The animal holder consists of a polystyrene 50 mL conical tube with the conical end cut open to allow for gas flow. The cap is modified with a square hole for attaching the axle to the motor. The mouse is oriented in the holder with its head toward the conical end, and the holder is placed on the 3D-printed stage (**B**). The assembled system is placed inside the imaging chamber with the motor wired to a remote controller that is fed through a light-tight port on the side of the IVIS Lumina XRMS (**C**).

The basic components of the MiSpinner system are a step-motor attached to a customized 3-D printed stage, which stabilizes the animal holder during the rotation process (Fig. 1). The animal holder is a modified polystyrene 50 mL conical tube, with the conical end cut open to allow oxygen and anesthesia flow to the animal, and a screw cap modified with a square 8 × 8 mm hole punched in the center for connection to the step motor. A critical component is a custom-made foam cylinder with a central slit that is wrapped around the base of the mouse-tail to further stabilize the animal during the rotation process. We found that incorporating this foam cylinder greatly reduced animal movement during the rotation process. The assembled system is connected to the existing gas supply manifold within the IVIS Lumina XRMS system. Acquisition occurs step-wise, with the user pressing a button to rotate the animal a pre-programmed number of degrees, followed by image acquisition as normal and repetition of the process until the animal has rotated 360°.

### Multi-Angle BLI in Mouse Bearing Multiple Tumors

**Figure 2:**
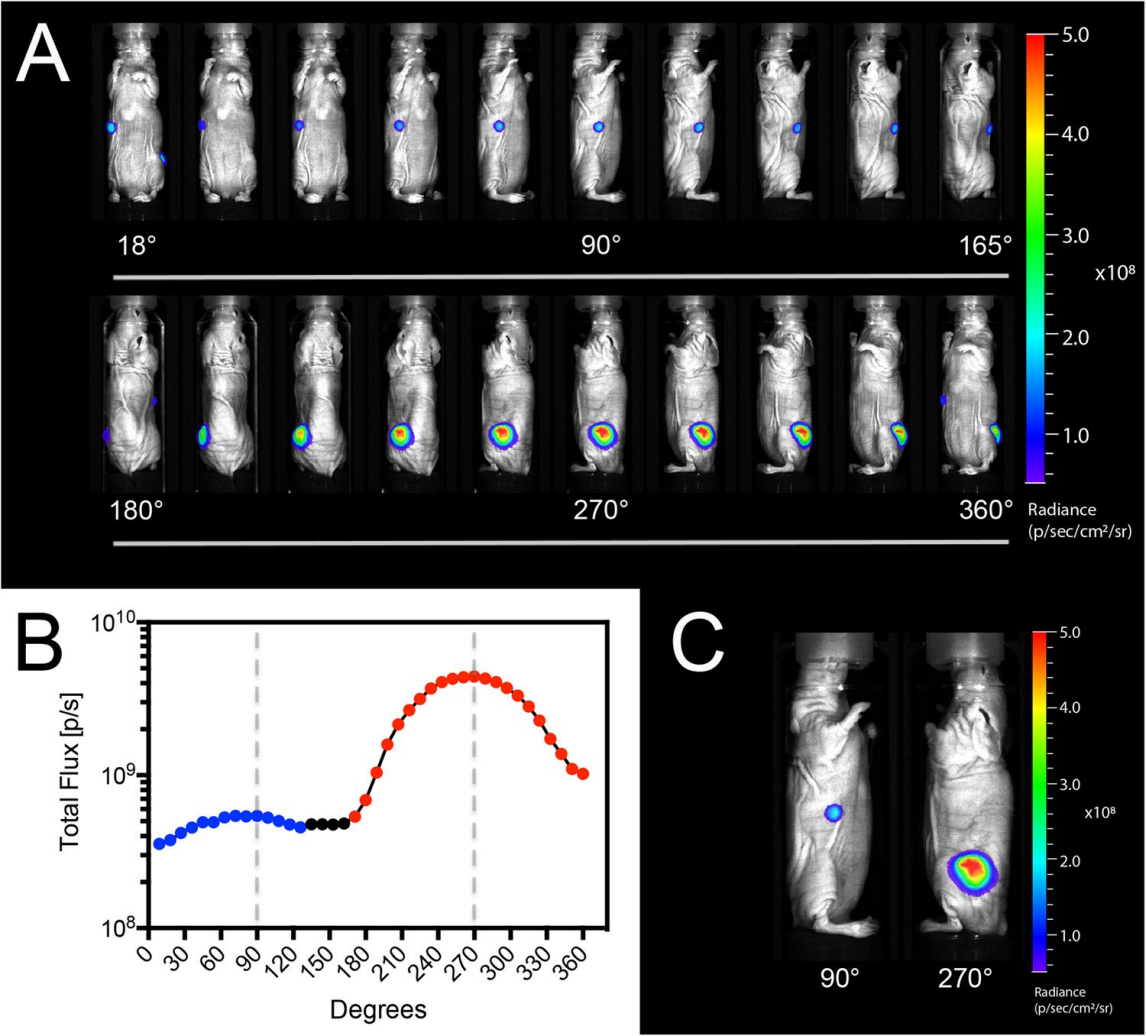
(**A**) A Mouse implanted with two opposing MDA-MB-231-Luc2-GFP tumors of different sizes imaged by BLI at 15° intervals by 360° surface mapping. (**B**) Total flux plotted against each respective angle of rotation. The 90° and 270° datasets signal were determined to be the maxima for each individual tumor and are designated by dashed lines on the plot. (**C**) Images for the 90° and 270° datasets determined to be the local maximas for total light production in panel B.

To simulate a mouse bearing a primary tumor and smaller distal metastasis, one NCR nu/nu female mouse was implanted with 2 × 10^6^ MDA-MB-231-Luc2-GFP cells in the left flank and 1 × 10^6^ cells in the right flank. This mouse was imaged using our MiSpinner system at 6 weeks post-implantation with 9° intervals for full 360° rotation, shown in Fig. 2A (images shown at 18° intervals). In Fig. 2B, bioluminescent flux is plotted against the degrees of rotation for each respective image. Two distinct peaks were resolved from this plot, with peak signal at 90° and 270° denoted by dashed lines corresponding to the images in Fig. 2C.

### Longitudinally Assessing Tumor Growth with Multi-Angle BLI

**Figure 3:**
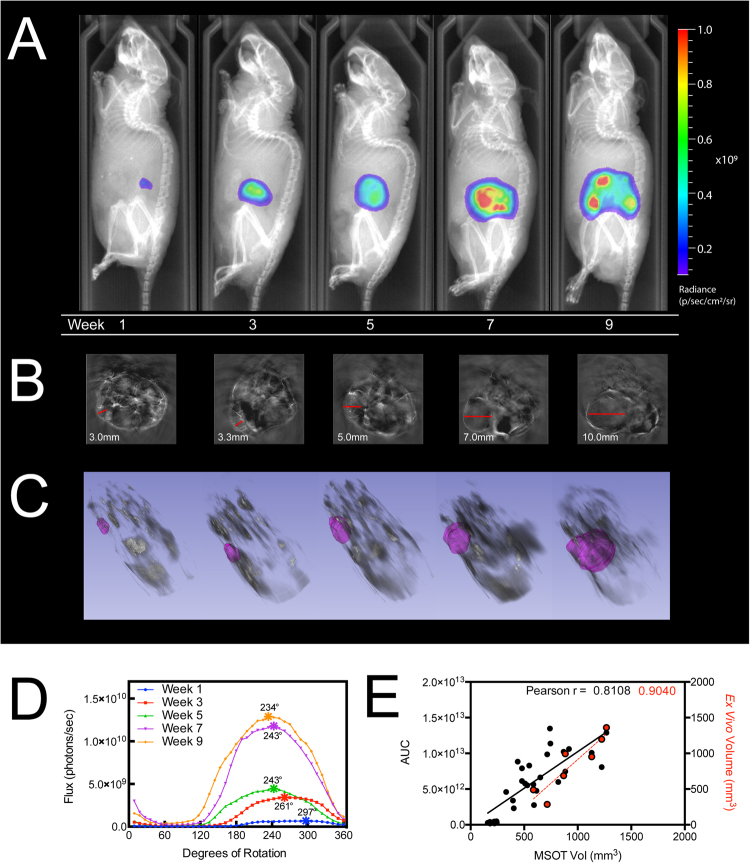
Subcutaneous PC-3-Luc2 implanted tumors longitudinally imaged using 360° BLI and MSOT. (**A**) MiSpinner-determined optimal angle of rotation (i.e. peak luminescent flux) overlaid on X-ray at bi-weekly time points. (**B**) Transverse sections of longitudinal MSOT scans at the site of tumor growth and (**C**) corresponding *in silico* segmentations superimposed on 3D rendering of whole-body MSOT scans with 850 nm background signal. (**D**) Luminescent flux is plotted against the relative degrees of rotation with respective peak angles denoted by (*). (**E**) Linear fit and Pearson correlation of MSOT segmented tumor volumes versus the area under the curve (*AUC*) of bioluminescent flux (solid black line) and *ex vivo* volumes (dashed red line) of mice (n = 7).

To demonstrate how surface mapping of BLI can be used to track longitudinal tumor growth, Fig. 3A shows one mouse imaged with the optimal imaging angle (i.e. peak luminescent flux), at bi-weekly time points, overlaid on X-ray. We also acquired MSOT scans at corresponding time points and determined tumor volumes *in silico* by segmentation (Fig. 3B,C). Figure 3D shows the longitudinal 360° flux curves with the optimal imaging angle denoted by an asterisk. In Fig. 3E, MSOT determined tumor volumes are plotted against both AUC and *ex vivo* volumes with corresponding linear fits (n = 7 mice). Corresponding Pearson correlation values are shown at the top, and comprehensive correlations are listed in Table 1.

**Table 1:** Pearson correlation of longitudinal caliper volumes and *in silico* volumes from MSOT segmentation with standard BLI, optimal angle BLI using the *MiSpinner*, and AUC in a PC-3-Luc2 subcutaneous tumor model. Endpoint *ex vivo* tumor volumes are shown with respective correlations to MSOT and caliper-determined tumor volume. *p* values are two-tailed with alpha equal to 0.05.

	Parametric correlation (Pearson r)	
	MSOT Volume	Caliper Volume
AUC (n = 30)	0.8108 (*p* < 0.0001)	0.7930 (*p* < 0.0001)
Optimal angle BLI (n = 30)	0.8073 (*p* < 0.0001)	0.7938 (*p* < 0.0001)
Standard BLI (n = 30)	0.7551 (*p* < 0.0001)	0.7380 (*p* < 0.0001)
MSOT volume (n = 30)	n/a	0.9270 (*p* < 0.0001)
Caliper volume (n = 30)	0.9270 (*p* < 0.0001)	n/a
*Ex vivo* (n = 7)	0.9040 (*p* = 0.0052)	0.7642 (*p* = 0.0455)

### Multi-Angle BLI Correlates More Significantly with Tumor Volume than Standard BLI

To assess multi-angle BLI’s utility for defining longitudinal tumor growth, we undertook an expanded study with n = 7 mice. Figure 3E demonstrates MSOT-determined tumor volumes’ correlation with AUC. *Ex vivo* volumes are represented in red and plotted against MSOT volumes with fit denoted by the red dashed line. Table 1 summarizes the results of standard BLI, MiSpinner-determined optimal angle BLI, and AUC linear correlations to caliper and MSOT volumes. Notably, MSOT-determined volumes strongly correlate with *ex vivo* volume measurements (Pearson *r* = 0.904; *p*-value of 0.005); as expected, caliper volumes poorly correlate with *ex vivo* volumes (Pearson *r* = 0.764; *p*-value of 0.046). AUC best correlates with both caliper and MSOT volumes, but this correlation is only slightly higher than optimal-angle BLI. Finally, standard BLI had the weakest correlation with caliper and MSOT volumes.

### Demonstration of Positional Bias using Multi-Angle BLI

As shown in Supplemental Fig. 1, we imaged a multi-lobular tumor at 7.5° intervals around a 360° centralized axis. The figure shows images at 15° intervals to demonstrate the difference in overall signal intensity at each orientation. Qualitatively, signal appearance varies drastically with minor changes in orientation. Supplemental Fig. 1B further illustrates the differences in luminescent signal morphology. At 90°, the tumor appears to be a single large lobe with a potentially smaller lobe slightly superior to the major lobe. However, at 135° the tumor morphology indicates two distinct major lobes plus a small third lobe. To quantitate the differences in signal, four regions of interest (ROI) were arranged in quadrants centered on the tumor region. The resulting flux data for each quadrant and the sum of all quadrants are plotted against the relative degrees of rotation in Supplemental Fig. 1C, in which dashed lines correspond to the 90° and 135° images in Supplemental Fig. 1B. In the sum of all quadrants, we observed two distinct peaks of signal that correspond with the two major lobes. The multi-lobular signal morphology in this tumor revealed the optimal angle to be 135°, as this is where the signal is most evenly dispersed across the quadrants.

## Discussion

BLI is a powerful tool for preclinical cancer models but longitudinal monitoring of distal metastasis by BLI can be challenging without a standardized means to acquire consistent images between time points. Due to the scattering effects of light in tissue, bioluminescent and fluorescent signal intensity can vary significantly due to animal positioning. Therefore, collecting data from multiple vantage points far more accurately defines optical sources within an animal. Ultimately, this positional bias can often produce diffuse bioluminescent signal measurements that necessitate increased animal numbers to achieve power in a given study.

The concept of imaging multiple angles to reconstruct original targets is not novel; indeed, this is the rationale underpinning imaging modalities, such as fluorescence molecular tomography (FMT), which can 3D-localize fluorescent reporters^26–28^. By contrast, diffuse light imaging tomography (DLIT; PerkinElmer Inc.), which acquires luminescence at different wavelengths, uses a sequence of filter sets to approximate depth within a pseudo-3D space^29^. One significant difference between FMT and DLIT for detection of distal sites of metastases in pre-clinical models is that there is greater interference from tissue at standard BLI wavelengths as compared to more near-infrared FMT wavelengths. To rectify this recent advances in bioluminescence tomography (BLT) have also been explored using a gantry-type rotating scaffold and multiple cameras that move around a static animal holder^30^. Multi-detector BLI systems have been engineered to simultaneously image from multiple angles and attempts to reconstruct those images to develop true BLT datasets. Those systems are custom builds and not readily available to other academic labs. Other newer imaging systems that have tried to overcome the positional bias include the InSyTe FLECT (Northridge Tri-Modality Imaging Inc.), which uses a static animal stage and a 48-detector ring that rotates around the stage to collect 360° tomographic optical data. To date, we found no published data that employ this technology. Our novel device is designed to bridge this gap and easily fit into many standard-imaging systems with top-mounted camera configurations making it immediately implementable to a broad user base. Our findings suggest that datasets collected by actuated rotation of an animal may provide a fast initial assessment of disease progression and allow for oversampling of a given region in optical tomography (FMT and BLT) studies, thereby improving the quality of reconstruction images allowing for the detection of secondary metastasis sites and overall assessment of tumor geometry.

Our *MiSpinner* system uses a cylindrical polystyrene animal holder that fits snugly around the animal and is further stabilized by a foam insert that is wrapped around the tail base. This approach results in consistent positioning across time points in longitudinal studies. Further, this animal holder design minimizes movement during the rotation process while maintaining a central axis. For the purposes of this study, we imaged only one mouse at a time; however, there is sufficient space within most top-mounted imaging systems’ field of view for multiple mice to be imaged simultaneously using this method, thereby increasing the eventual throughput of small animal imaging using 360° surface mapping of bioluminescent signal.

The results of this study demonstrate that net bioluminescent signal can vary with even relatively minor animal rotation, and, as a result, we sought to define the maximal BLI signals generated by the tumors in these animals over time. Figure 3A shows tumor growth progression as assessed by multi-angle BLI at bi-weekly time points with the optimal angle of rotation shown for each. Close examination of the X-ray data suggests the angle of maximum flux is consistent during the early stages of tumor progression (weeks 1–5). However, once the tumor advances in size, the angle with peak luminescent signal alters noticeably. We believe this change is attributable to increased tumor burden (weeks 7–9). By week 9, the tumor is likely undergoing necrosis that contributes to the diffuse distribution of optical signal relative to the skeletal landmarks.

With a larger cohort (n = 7), we monitored tumor progression longitudinally using standard BLI and 360° multi-angle BLI, with volumes monitored by traditional caliper measurements and segmentation from MSOT scans. We show that MSOT segmented volumes correlated with endpoint *ex vivo* volumes with a high degree of significance (Pearson *r* = 0.9040; *p*-value of 0.0052), while caliper volumes correlated poorly in comparison (Pearson *r* = 0.7642; *p*-value of 0.0455). Of note, there was an almost 10-fold reduction in the *p*-value, and for the caliper measurements, these correlations approached non-significant limits. As a result, we found better correlation of MSOT segmented tumor volumes with either standard or 360° BLI datasets compared to caliper-derived volumes. Therefore, we adopted MSOT as our primary standard for determining tumor volumes for correlating measures of bioluminescent signal, secondary to traditional caliper measurements.

As should be expected, we also noticed that both the height and breadth of luminescence signal increased with tumor growth. These increases made it necessary to also examine the integral of the signal or the area under the curve (AUC). We found that while the optimal-angle BLI had better correlation with tumor volumes as compared to standard 2D BLI (Pearson *r* = 0.8073 and 0.7551 respectively, with *p*-value < 0.0001 for both), correlations were further improved using the AUC measures (Pearson *r* = 0.8108, *p*-value < 0.0001). While the improved correlation of AUC versus optimal-angle BLI was modest, we believe the AUC measurements may be less prone to error and may thus provide a more reliable means of assessment. With the paradigm of cancer research shifting towards orthotopic and metastatic models, multi-angle BLI may be a useful tool for localizing metastasis and assessing tumor growth and regression in the preclinical setting.

In summary, by incorporating the *MiSpinner* device we minimized positional bias by standardizing animal placement during a longitudinal study and demonstrated the ability to resolve and quantify signal from multiple tumors within the same animal. Developing this device and protocol allowed us to determine the maximum BLI values for a given tumor at a given time, and thus significantly improved BLI correlation with both caliper and photo-acoustic measures of tumor volume. Given the broad potential for using the *MiSpinner* in various currently available top-mounted single detectors, we believe this advancement has the potential to improve assessment of tumor growth and therapeutic regression in relevant cancer models, particularly in academic labs.

## Materials and Methods

### Animals and Tumor Model

All animal procedures were designed in accordance with the Guide for the Care and Use of Laboratory Animals of the National Institutes of Health and approved by the Institutional Animal Care and Use Committee of Auburn University under protocols AU-2015-2808 and AU-2012-2144. Xenografts of luciferase-expressing human prostate cancer cells (PC-3-Luc2 Bioware Ultra, PerkinElmer Inc., Waltham, MA) or breast cancer cells (MDA-MB-231-Luc2-GFP Bioware Ultra) were established subcutaneously in NCr nude, 6–8 week-old, male or female mice (Taconic Biosciences Inc., Albany, NY), respectively. In brief, cells, cultured routinely in F-12K medium (Mediatech Inc., Manassas, VA) or DMEM:F12 medium (HyClone Laboratories Inc., Logan, UT) and supplemented with 10% (v/v) fetal bovine serum (HyClone Laboratories Inc.), were harvested at sub-confluence using 0.25% (v/v) trypsin (Mediatech Inc.) and collected as a suspension with complete medium. Total cells were counted and then were centrifuged at 250 × *G* for five minutes to pellet the cells. Pelleted cells had media removed by aspiration and subsequently were resuspended in phenol-free and serum-free F-12K media to produce a final concentration of 1 × 10^7^ cells per mL. Prior to injection, the cell suspension was mixed (1:1, v/v) with ice-cold Matrigel (BD Biosciences, San Jose, CA). While providing 1–3% isoflurane gas (Henry-Schein, Melville, NY) with oxygen to the mice, a 1.0 mL syringe with a 26-gauge needle (BD Biosciences) was used to implant 200 µL of cell mixture (1 × 10^6^ cells) subcutaneously into the left flank of each mouse. In total, 16 mice were used for this study. For the multi-tumor mouse study shown in Fig. 2, one female NCR-Nu mouse was implanted in the left flank with 2 × 10^6^ MDA-MB-231-Luc2-GFP cells (200 uL) and 1 × 10^6^ cells (100 uL) in the right flank. Cells were prepared using adjusted concentrations and implanted using the procedure outlined above.

### MiSpinner Modified Imaging System for Bioluminescence Measurement

The MiSpinner prototype is comprised of a stepper motor, remote controller, and an animal holder that is connected to the standard gas anesthesia system within the imaging chamber. A universal valve actuator (Valco Instruments Company, Inc., Houston, Texas) was selected as the motor and controller for 2 reasons: (1) the actuator can precisely control the degree of rotation between steps as these motors are used normally to switch between valve positions in liquid chromatography systems and (2) the actuator, unlike other simple stepper motors, had to be durable enough and impart enough torque to withstand the radial force imparted by the mouse and animal holder. Prior to imaging, the motor can be programmed to turn a defined number of precise iterations within 360°. The stage connected to the motor was custom designed using 123D Design v11.2.1 (Autodesk Inc., Mill Valley, CA) and 3D printed. The animal holder is designed for single use to prevent cross-contamination and consists of a modified polystyrene 50 mL conical tube without obvious markings (Denville Scientific, South Plainfield, NJ). The screw cap of the conical tube was punched with a centered 8 × 8 mm square hole for attachment to the motor axle. The conical end of the tube serves as a nose cone, with the end cut open to allow gas exchange. Mice were inserted into the animal holder with the head oriented toward the conical end and stabilized by a foam cylinder wrapped around the tail base. The motor and animal holder were placed into the imaging chamber of the *IVIS Lumina XRMS* (PerkinElmer Inc.) and connected remotely to the controller and power supply on the outside of the chamber via data and power cords fed through a light-tight port. Figure 1 shows both the MiSpinner system inside the imaging chamber of the *IVIS Lumina XRMS* and the controller outside the chamber.

### Tumor Volume Determination from Digital Caliper Measurements

Tumor growth was assessed three times per week using digital caliper measurements of tumor x/y dimensions. Prior to imaging sessions, mice received anesthesia using 1–3% isoflurane gas with medical-grade oxygen while three independent reviewers measured tumor dimensions to determine a mean volume as a basis for correlation. Volumes were calculated using a standard formula derived from the volume of an ellipsoid,

1 $${Caliper}\,Tumor\,Volume=(\pi /6)\,\bullet \,(larger\,diameter\,Y)\,\bullet \,{(smallerdiameter)}^{2}$$

Humane endpoints were defined as tumors that approached or exceeded 1500 mm^3^. Otherwise, tumors were monitored for 9 weeks before mice were euthanized. Tumors were resected and then measured relative to three axial dimensions of diameter, and volumes were calculated using

2 $$Volume=(\pi /6)\,\bullet \,(diameter\,X)\,\bullet \,(diameter\,Y)\,\bullet \,(diameter\,Z)$$

### MSOT Acquisition and In Silico Tumor Volume Determination

Prior to imaging, all mice were anaesthetized with 1–3% isoflurane and medical-grade oxygen. MSOT imaging was performed using the *InVision 256-TF* and *ViewMSOT* v3.6.0.119 (iThera Medical GmbH, Munich, GE)^31^. Briefly, a tunable optical parametric oscillator (OPO) pumped by an Nd:YAG laser provides excitation pulses for 9 ns at wavelengths of 715–850 nm at a repetition rate of 10 Hz with a wavelength tuning speed of 10 ms and a peak pulse energy of 100 mJ at 730 nm. Ten arms of a fiber bundle provided even illumination around the subject with a relative bandwidth of 8 mm. Our MSOT version has 256-transducers, with a center frequency of 5 MHz (60% bandwidth), organized in a concave array of 270-degree angular coverage and a 4 cm curvature radius. The in-plane resolution is dependent on the distance from the center of rotation and varies from 150 μm in the center to 550 μm at a distance of 1 cm. The minimally achievable slice thickness is 800 μm and the physical slice thickness is approximately 800 μm according to the focal zone of the ultrasound detector. By oversampling the slice thickness, image quality can be improved for optimal 3D reconstruction. For the purposes of this study, scans were performed using a 0.5 mm slice separation spanning the entire tumor region for each mouse, allowing oversampling of approximately 0.3 mm to ensure adequate reconstructed image quality. A tunable laser provides excitation light at 715, 730, 760, 800, and 850 nm. Wavelengths were selected to differentially evaluate the contributions of endogenous chromophores to the overall photo-acoustic signal with 850 nm as an anatomical reference. Photo-acoustic signals from chromophores parallels their absorbance spectra. The relative contribution of different chromophores to the overall photo-acoustic signal can be evaluated by linear un-mixing of datasets from specific excitation compared to the absorbance spectra of the chromophores. At these standard settings, the MSOT can separate oxygenated from deoxygenated hemoglobin. This is possible due to the broad singular absorbance peak of the deoxygenated hemoglobin compared to bifurcated peak of oxygenated hemoglobin causing distinctly different absorbance values over 650–750 nm for these endogenous chromophores.

Slices from each scan were exported from *ViewMSOT* (iThera Medical GmbH) to *ImageJ* v2.0.0-rc-15/1.49 h (https://imagej.nih.gov/ij) and then exported as a stack of TIFF images. Tumor segmentation and *in silico* volume determinations (Fig. 3B) were performed using *3D Slicer* v4.5.0 (https://www.slicer.org) with the *Editor*, *Models*, and *Volume Rendering* tools, respectively. In brief, TIFF stacks of each MSOT scan were loaded into 3D Slicer and image spacing was set to the acquired dimensions of 0.075 mm × 0.075 mm × 0.5 mm. Under the *Editor* module, a “new structure” was added and the “paintbrush” tool was used to define the margins of the tumor in each slice. Once complete, the “merge and build” function was used to reconstruct the segmented volume. The *Model* module was used to calculate *in silico* volumes by multiplying the surface area of each segmented slice by the slice distance over the whole of each dataset. The *Volume Rendering* tool was used to overlay the segmented tumor volumes created in the *Model* module over a back-projection of each corresponding TIFF stack dataset.

### Multi-Angle BLI of Mouse Bearing Multiple Tumors

One female mouse was implanted with 2 × 10^6^ MDA-MB-231-Luc2-GFP cells in the left flank and 1 × 10^6^ cells in the right flank to simulate primary and metastatic tumor growth. This mouse was imaged for bioluminescence at 4 weeks following implantation using 9° intervals around 360°, shown in in Fig. 2A (18° intervals are shown). The mouse was anaesthetized with medical-grade oxygen supplemented with 1–3% isoflurane and then injected intraperitoneally (IP) with 500 μL of 30 mg/mL *D*-luciferin (600 mgs/kg dose for a standard 25-gram mouse; PerkinElmer Inc., Waltham, MA) and allowed to distribute for 20 minutes prior to imaging. Bioluminescent images were acquired using 3-second exposures and overlaid on corresponding photographs. All images were loaded in *Living Image* as a batch sequence for simultaneous thresholding of luminescent signal in each acquired image. ROI were measured by the rate of photons reaching the CCD (*flux*), determined using static rectangular ROI (9 × 4 cm) placed in the exact location around the animal across all rotation degrees. Bioluminescent flux from each ROI was plotted against the relative degrees of rotation for each respective dataset, shown in Fig. 2C. Images with peak bioluminescent flux for each respective tumor are shown in Fig. 2B and denoted by dashed lines in Fig. 2C.

### Longitudinal Multi-Angle *In Vivo* BLI

Prior to each imaging session, three mice were chosen for a kinetic assay to determine the enzyme saturation time point, i.e. the optimal imaging window during which luciferases are saturated with luciferin, as evidenced by a plateau in luminescent signal. Mice were anaesthetized with medical-grade oxygen supplemented with 1–3% isoflurane and then injected intraperitoneally (IP) with 500 μL of 30 mg/mL *D*-luciferin (600 mgs/kg dose for a standard 25-gram mouse; PerkinElmer Inc., Waltham, MA). Kinetic assays were performed with standard bioluminescence images acquired every 30 seconds for a period of 45 minutes. The following day, mice were injected IP with 500 μL of 30 mg/mL *D*-luciferin. Standard BLI was performed at the beginning of the saturation point and immediately followed by 360° BLI using the *MiSpinner* with 15° intervals (24 images total). Exposure time varied from 1–5 seconds (depending on total counts) with small binning and subject height set to 4.0 cm. The data automatically converted into calibrated radiance by *Living Image* for standardization and comparison.

Additionally, once per week throughout the 9-week time course, a single mouse was selected for BLI with X-ray performed at precise 7.5° turns (48 images) around 360°; the results are shown in Fig. 3A. X-ray was acquired using the high-resolution setting, 5-second exposure per image, and the scintillator swing-arm positioned to “large animal” to accommodate the *MiSpinner* system. The total radiation dose was 1–3mGy per X-ray image with the total exposure of 48–144 mGy per session, a level that encompasses the standard single CT dose of 120 mGy^32–34^. This exposure is assumed not to alter tumor progression as chromosomal aberrations in mammals have been reported only at 250–300 mGy. The exposure level is also well below the lethal single dose of X-ray radiation for a mouse; 50% of animals survive a lethal dose of 26,800 mGy (n = 28)^35^.

### 360° Bioluminescent Image Processing and Statistical Analysis

Following acquisition, all images in each multi-angle dataset were loaded in *Living Image* as a batch sequence for simultaneous thresholding of luminescent signal in each acquired image. ROI were measured by the rate of photons reaching the CCD (*flux*), determined using static rectangular ROI (9 × 4 cm) placed in the exact location around the animal across all rotation degrees and time points. Bioluminescent flux from each ROI was plotted against the relative degrees of rotation for each respective dataset. AUC and optimal BLI angle were determined for each dataset using the “area-under-the-curve” and “column statistics” functions in *Prism 6* (GraphPad Software Inc., La Jolla, CA). Correlations among caliper-determined tumor volumes, MSOT *in silico*-determined tumor volumes, AUC, optimal Angle BLI, and standard BLI were assessed using parametric Pearson correlation in *Prism 6*. All *p*-values listed are two-tailed with alpha equal to 0.05. *A solid black line for each respective correlation plot denotes a linear fit model*. *Ex vivo* red circles indicate volumes, and the respective linear fits are shown by dashed red lines. Six mice with implanted tumors that failed to grow beyond a 700 mm^3^ threshold after 9 weeks were excluded from this study. Also excluded was a mouse possessing a highly irregular tumor morphology and a mouse that died shortly after the implantation procedure.

### Analysis of a Multi-Lobular Tumor Signal Using Multi-Angle BLI

During preliminary studies, a mouse with a multi-lobular PC-3-Luc2 tumor was selected for multi-angle BLI to assess differences in signal detected at different angles at a single point in time. BLI was performed at precise 7.5° intervals around 360° (48 images total). In Supplemental Fig. 1, square ROI of equal quadrants were centered on the tumor region. The photon flux for each individual quadrant and the sum of all quadrants at each orientation were plotted against their respective degrees of rotation.

### Video Production

Video files (*Supplemental Figures*) were compiled by loading all images from each 360° dataset into a sequence and equalizing the parameters and scale across all frames using *Living Image* v4.4.17106 (PerkinElmer Inc.). All frames were exported from *Living Image* as TIFF files. Next, images were loaded into Adobe Photoshop CS6 (v13.0.6) as sequential image files (via *File-Scripts-Load Files into Stack*), cropped, arranged, and exported as “.mov” files.

## Electronic supplementary material

Supplementary Figure 1

Supplementary Figure 2

Supplementary Figure 3

Supplementary Figure 4
